# Microbial carbon source utilization in rice rhizosphere and nonrhizosphere soils with short-term manure N input rate in paddy field

**DOI:** 10.1038/s41598-020-63639-8

**Published:** 2020-04-16

**Authors:** Tang Haiming, Xiao Xiaoping, Li Chao, Pan Xiaochen, Cheng Kaikai, Li Weiyan, Wang Ke

**Affiliations:** Hunan Soil and Fertilizer Institute, Changsha, 410125 P. R. China

**Keywords:** Ecology, Environmental impact

## Abstract

Carbon (C) plays a vital role in regulating soil nutrient cycling and increasing soil microbial community, but there is still limited information on how C source utilization characteristics responds to soil physical and chemical properties changes under double-cropping rice (*Oryza sativa* L.) paddy field in southern China. Therefore, the effects of different short-term manure nitrogen (N) input rate managements on C source utilization characteristics in rice rhizosphere and non-rhizosphere soils under double-cropping rice field in southern China were studied by using ^18^O-H_2_O method. Therefore, a field experiment were established in Ningxiang city of Hunan Province, and five different fertilizer treatments were applied: (1) 100% N of chemical fertilizer (M0), (2) 30% N of organic manure and 70% N of chemical fertilizer (M30), (3) 50% N of organic manure and 50% N of chemical fertilizer (M50), (4) 100% N of organic manure (M100), and (5) without N fertilizer input as control (CK). The results showed that soil microbial biomass C content, soil microbial growth rate, and soil microbial basal respiration with application of organic manure treatments (M30, M50, M100) were significantly higher (*p* < 0.05) than that of CK treatment. And the soil C utilization efficiency with M0 treatment were significantly higher (*p* < 0.05) than that of M100 treatment. Compared with CK and M0 treatments, the metabolic capacity of soil microorganisms to exogenous C sources with M30, M50 and M100 treatments were increased. The largest types of exogenous C source was carboxylic acids, followed by amino acid and carbohydrate, and complex compounds was the smallest. The RDA analysis results indicated that fertilizer treatments significantly changed the utilization characteristics of soil microorganisms to exogenous C sources. As a result, this study found that characteristics of soil C source utilization were significantly affected by different short-term manure N input rate managements.

## Introduction

Soil microorganisms play a vital role in regulating soil physical and chemical properties, increasing soil microbial community and diversity, maintaining soil quality and soil fertility, and contributing to crop growth^1^. Soil organic carbon (SOC) content were strongly affected by soil microorganisms^2^. And rhizosphere processes were important for the functioning of paddy ecosystems for that rhizosphere processes were regulated nutrient cycling, such as soil organic matter (SOM) decomposition and nutrient dynamics, which were closely related to crop root and their related rhizosphere processes^3^. Meanwhile, the non-rhizosphere processes were also play an important role in biogeochemical cycling and crop growth. The soil microbial carbon (C) use efficiency (CUE) were usually considered as the proportion of growth and organic C taken up in organic C, and it was important comprehensive characteristics of soil microbial community metabolism^4,5^. In the previous studies, it was found that types of exogenous carbon sources mainly utilized by soil microorganism were included carboxylic acids, amino acid, carbohydrate, complex compounds, and so on^6,7^. Although the soil microbial community metabolism was important for C cycling, there is still limited information on the ecophysiology of microbial C cycling and the effects of different factors on shape soil microbial CUE^8^.

Soil microbial CUE were affected by different agriculture managements, such as the crop types, tillage, fertilizer regime, irrigation patterns, and so on. Soil biogeochemical characteristics and nutrient cycle were changed under application of different fertilizer conditions, which in return change soil microbial biomass stoichiometry and microorganisms^9^. As a result, soil microbial CUE were affected by taken different fertilizer practices. Manzoni *et al*.^4^ results showed that soil microbial CUE achieved maximum with low C:N ratio. Meanwhile, SOC concentration and quality, microbial community compositions were also major factors in changing soil microbial CUE and soil microbial biomass content^10^. In the previous studies, it was found that CUE of soil microbial communities were closely related with decomposing complex compounds, respiration rate of unit C assimilation^11^. Don *et al*.^12^ results indicated that organic C mineralization were decreased with decrease of C concentration. Tang *et al*.^13^ research found that C source utilization of soil microorganisms in paddy field were increased under combined application of organic manure or crop residue with inorganic fertilizer conditions. Fang *et al*.^14^ results showed that soil microbial biomass and C-use efficiencies were increased by applied with organic amendment and inorganic fertilizer. But other study indicated that soil microbial CUE were decreased under combined application of plant residue with inorganic fertilizer conditions^15^. Therefore, soil microbial CUE were closely related to soil organic C quality and contents. Furthermore, some studies indicated that soil microbial CUE of fungi were higher than that of bacteria^10^. However, other studies indicated that there was no significant difference in soil fungal and bacterial CUE^16^.

The method for investigated soil microbial CUE in paddy ecosystem were accepted by more and more researchers^5^. Previous studies showed that soil microbial CUE were calculated by soil microbial incorporation and respiration of specific ^13^C-labeled substrates^17^. However, this method found it was very different soil microbial CUE estimates for that this determination method confuses soil microbial CUE with the specific substrate^5^. To overcome these problems, a novel method was suggested based on the incorporation of ^18^O from water into DNA during soil microbial biomass growth. That is, during the incubation time, the increase of soil microbial biomass C was investigated according to the ^18^O-DNA^9^. Therefore, the soil microbial CUE were calculated according to both the soil microbial growth rate and the basal respiration rate.

Rice (*Oryza sativa* L.) is one of the main crops in Asia, and double-cropping rice system (early rice and late rice) is the main land use in southern China^18^. It is benefit practices for maintain the paddy soil quality and fertility by application of fertilizer managements (organic fertilizer, inorganic fertilizer, and so on). And the manure N input rate managements may profound effects on soil physical and chemical characteristics such as pH, soil bulk density, SOC content, which in return affect soil microbiological properties and characteristics of carbon utilization. We hypothesized that: (i) soil microbial CUE were increased under application chemical fertilizer conditions; (ii) metabolic capacity of soil microorganisms to exogenous carbon sources would be higher with organic manure input conditions than that of with chemical fertilizer and without N fertilizer input conditions. Therefore, a short-term field experiment with different fertilizer treatments were conducted in a double-cropping rice system in the southern China. Hence, the objective of this study was (1) to investigate the microbial CUE in rice rhizosphere and non-rhizosphere soils under different manure N input rate fertilization conditions; (2) to evaluate metabolic capacity of soil microorganisms to exogenous carbon sources with different manure N input rate practices in a double-cropping rice system.

## Results

### Soil microbial carbon utilization efficiency

**Figure 1:**
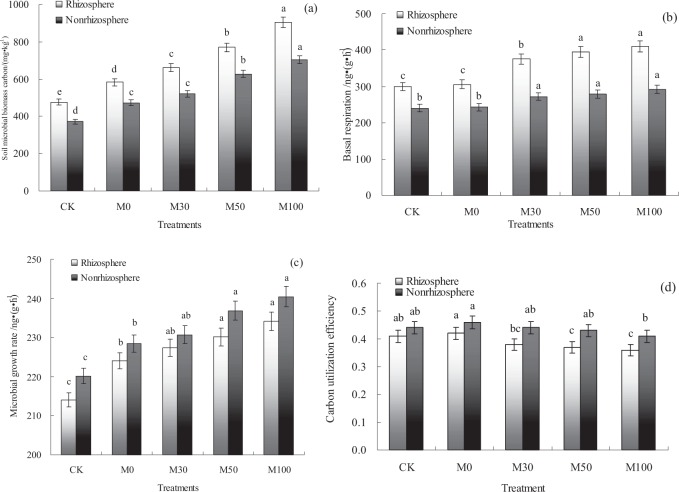
Soil microbial distribution in rhizosphere and non-rhizosphere soils with different fertilizer treatments. M0: 100% N of chemical fertilizer, M30: 30% N of organic manure and 70% N of chemical fertilizer, M50: 50% N of organic manure and 50% N of chemical fertilizer, M100: 100% N of organic manure, CK: without N fertilizer input as a control. (**a**) is soil microbial biomass carbon, (**b**) is basal respiration of soil microorganism, (**c**) is soil microbial growth rate, (d) is soil microbial carbon utilization efficiency. Error bars represent standard error of mean. Different letters are significantly different at *P* < 0.05 level. The same as below.

The effects of different manure N input rate treatments on soil microbial carbon utilization efficiency (CUE) in the double-cropping rice paddy field were shown in Fig. 1. The results showed that range of soil microbial biomass carbon (MBC) content was 371.61 to 904.83 mg kg^−1^ in rhizosphere and non-rhizosphere soils with different fertilizer treatments. And the soil MBC content in rhizosphere soils were higher (*p* < 0.05) than that of non-rhizosphere soils with different fertilizer treatments. In rhizosphere and non-rhizosphere soils, the lowest soil MBC content were observed with the CK treatment, while the highest soil MBC content were observed with the M100 treatment. The results showed that soil MBC content with M50 and M100 treatments were higher (*p* < 0.05) than that of M0 and CK treatments in the double-cropping rice paddy field (Fig. 1a). Compared with application of chemical fertilizer and without N fertilizer input practices, the soil MBC content were increased with application of organic manure practices.

The range of soil microorganism basal respiration was 240.32 to 410.36 ng (g·h)^−1^ in rhizosphere and non-rhizosphere soils with different fertilizer treatments. And the basal respiration of soil microorganism in rhizosphere soils were higher (*p* < 0.05) than that of non-rhizosphere soils with different fertilizer treatments. In rhizosphere and non-rhizosphere soils, the basal respiration of soil microorganism with M30, M50 and M100 treatments were higher (*p* < 0.05) than that of M0 and CK treatments, but there was no significant difference (*p* > 0.05) in basal respiration of soil microorganism between M50 and M100 treatments (Fig. 1b).

The soil microbial growth rate (C_Growth_) were calculated based on the equation as mentioned above. The results showed that range of C_Growth_ was 214.06 to 240.47 ng (g·h)^−1^ in rhizosphere and non-rhizosphere soils with different fertilizer treatments. And the C_Growth_ in non-rhizosphere soils were higher (*p* < 0.05) than that of rhizosphere soils with different fertilizer treatments. In rhizosphere and non-rhizosphere soils, the C_Growth_ with M50 and M100 treatments were higher (*p* < 0.05) than that of M0 and CK treatments, but there was no significant difference (*p* > 0.05) in C_Growth_ between M50 and M100 treatments (Fig. 1c).

The range of soil microbial CUE were 0.36 to 0.46 ng (g·h)^−1^ in rhizosphere and non-rhizosphere soils with different fertilizer treatments. And the CUE of soil microbial in non-rhizosphere soils were higher (*p* < 0.05) than that of rhizosphere soils with different fertilizer treatments. In rhizosphere soils, the CUE of soil microbial with M0 and CK treatments were higher (*p* < 0.05) than that of M50 and M100 treatments. In non-rhizosphere soils, the CUE of soil microbial with M0 treatment were higher (*p* < 0.05) than that of M100 treatment, but there was no significant difference (*p* > 0.05) in CUE of soil microbial among M0, M30, M50 and CK treatments (Fig. 1d).

### Characteristics of utilization of different types of exogenous carbon sources

**Figure 2:**
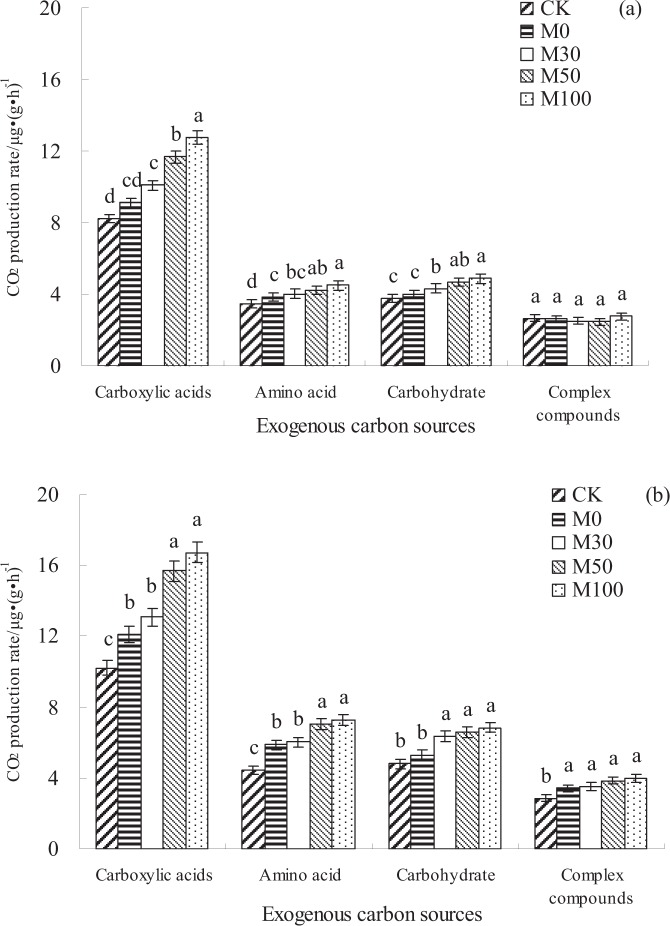
Characteristics of utilization of different types of exogenous carbon sources by rhizosphere and non-rhizosphere soils microorganisms with different fertilizer treatments. (**a**,**b**) indicate rhizosphere and non-rhizosphere soils, respectively.

The metabolic capacity of soil microorganisms to the types of exogenous carbon sources (carboxylic acids, amino acid, carbohydrate, complex compounds) in non-rhizosphere soils were higher (*p* < 0.05) than that of rhizosphere soils with different fertilizer treatments (Fig. 2). Compared with CK and M0 treatments, the metabolic capacity of soil microorganisms to exogenous carbon sources in rhizosphere soils were increased with application of organic manure managements (M30, M50, and M100 treatments). In rhizosphere and non-rhizosphere soils, there was no significant difference (*p* > 0.05) in microbial utilization of complex compounds among different fertilizer treatments (M0, M30, M50, and M100 treatments) (Fig. 2). In different types of exogenous carbon sources, the average utilization rate of carboxylic acids were higher (*p* < 0.05) than that of complex compounds with different fertilizer treatments.

**Figure 3:**
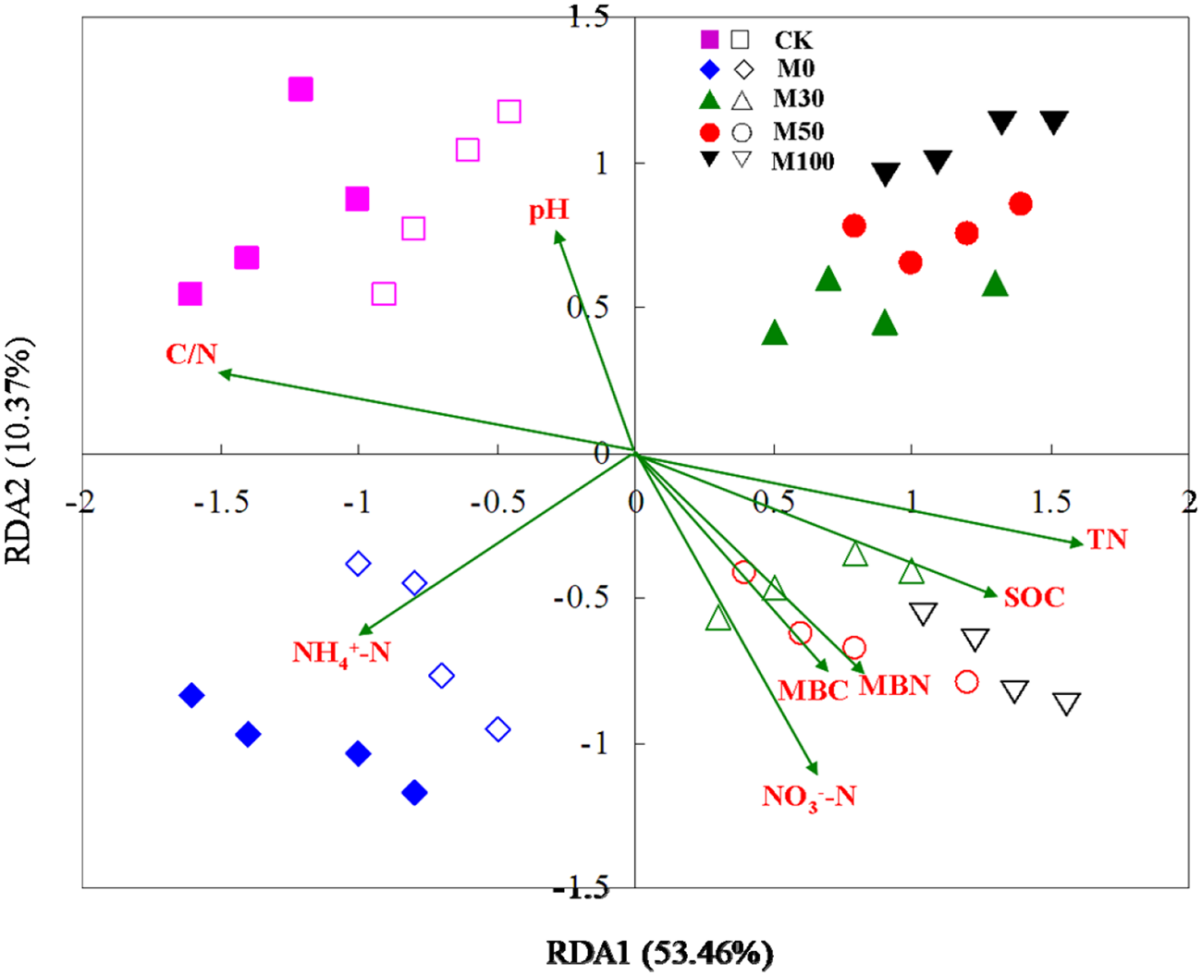
Redundancy analysis of microbial carbon source utilization rate and soil chemical properties. Solid icon and hollow icon indicate rhizosphere and non-rhizosphere soils, respectively. SOC: soil organic carbon, TN: soil total nitrogen, MBC: soil microbial biomass carbon, MBN: soil microbial biomass nitrogen.

Considering the whole soil microbial carbon source utilization rate, the results revealed there was a significant correlation between soil microbial carbon source utilization rate and soil chemical properties (Fig. 3). In addition, the soil chemical properties can explain the variation (53.46%) in soil microbial carbon source utilization rate between the M0, M30, M50, M100 and CK treatments. Under different fertilizer treatments, M0 and CK treatments were separated from application of organic manure treatments (M30, M50 and M100 treatments), indicating that fertilizer treatments significantly changed the utilization characteristics of soil microorganisms to exogenous carbon sources. The soil chemical properties was significantly correlated with the utilization characteristics of soil microorganisms to exogenous carbon sources including the C/N, NH_4_^+^-N, NO_3_^−^-N, SOC, and TN contents.

## Disscussion

In the present study, the results indicated that soil MBC content in rhizosphere and non-rhizosphere soils were increased with application of organic manure practices, compared with without N fertilizer input practices. The reason maybe that soil available nutrients increased by application of organic materials, which provided carbon substrates and nutrients for the growth and reproduction of soil microorganisms, and then increased soil microbial growth rate and microbial biomass^19^, compared with without N fertilizer input practices. Meanwhile, the soil MBC content with organic manure treatments were higher than that with chemical fertilizer alone treatment, the reason maybe that decomposition of organic manure, soil C/N and slow-acting nutrients were increased under input organic manure conditions, which promote the growth of soil microorganisms^20^. And the basal respiration of soil microorganism were increased under application of organic manure conditions, the reason maybe that decomposable organic carbon is the main carbon source for microbial utilization, and there are significant differences in the content of decomposable organic carbon under different manure N input fertilizer treatments^21^. On the other hand, rice root exudates can induce a positive stimulating effect of soil organic carbon decomposition, which in return increases soil microbial respiration^22^. And the amount of root exudates were affected by application of different fertilization practices, root biomass and root exudates were increased under application of organic manure conditions^9^. In this studies, the C_Growth_ with fertilizer treatments were higher (*p* < 0.05) than with without N fertilizer input treatment, the reason maybe that there are significant differences in soil organic carbon content among different fertilizer treatments, which may lead to significant differences in available carbon sources of soil microorganisms^23^.

In this study, the results showed that CUE of soil microbial in rhizosphere soils were lower than that of non-rhizosphere soils, the reason maybe that value of C_Respiration_ were higher in rhizosphere soils, thus the distribution of respiration carbon by rhizosphere soils microorganisms were higher than that of growth carbon. On the other hand, carbon content in rhizosphere soils were increased in the process of root system absorbs nutrients, and the soil microbial CUE were decreases with the increase of C content^9^. There is no significant difference in CUE of non-rhizosphere soils among different fertilizer treatments, the reason may be that physiological environment of non-rhizosphere soils is relatively stable, which was consistent with previous research^24^. In this study, the higher CUE of soil microbial with chemical fertilizer alone treatment (M0) than that of with organic manure treatments (M30, M50 and M100) consistent with our Hypothesis 1 that soil microbial CUE were increased under application chemical fertilizer conditions (Fig. 1), the main reason was that soil carbon nutrient ratio and carbon dioxide (CO_2_) through overflow respiration were increased, soil carbon nutrient ratio was benefit to meet the nutritional needs of soil microorganisms^10^, and thus lower soil microbial CUE under application of organic manure conditions^9^. However, the oxidase involved in the degradation of aromatic compounds in N were inhibited, the energy demand of soil microorganisms were reduced with chemical NPK fertilizer input, therefore, the soil microbial CUE were increased under application of chemical fertilizer conditions^9^.

The characteristics of soil microbial communities on carbon metabolism can reflect the bioavailability and functional diversity of soil microorganisms^7^. The average CO_2_ production rate was usually used as an effective indicator of soil microbial activity, and also reflects the ability of soil microbial communities to utilize carbon sources^5^. In the present study, the metabolic capacity of soil microorganisms to exogenous carbon sources with application of organic manure treatments were higher than with chemical fertilizer alone and without N fertilizer input treatments, the reason maybe that organic manure contains a large number of living microorganisms and active organic carbon sources, and thus the metabolism of microorganisms to carbon sources were promoted when application of organic manure. As a result, the higher metabolism of exogenous carbon sources with organic manure treatments than that of with chemical fertilizer alone and without N fertilizer input treatments consistent with our Hypothesis 2 that metabolic capacity of soil microorganisms to exogenous carbon sources would be higher with organic manure input conditions than that of with chemical fertilizer alone and without N fertilizer input conditions. And the metabolic capacity of soil microorganisms to exogenous carbon sources with chemical fertilizer alone treatment were higher than without N fertilizer input treatment, indicating that soil C/N were increased, soil microbial activity and decomposition rate were decreased, and thus soil microbial metabolic ability to carbon source were decreased under without N fertilizer input conditions^19^. Our results indicated that average utilization rate of carboxylic acids were higher than that of complex compounds, which suggested that carboxylic acid carbon sources account for only a small proportion of soil dissolved organic carbon, but they are important energy sources for the growth and metabolism of soil microorganisms^25^. On the other hand, degradation of complex compounds requires the interaction of various extracellular enzymes, more carbon sources and energy were invested in the synthesis of extracellular enzymes during soil microorganisms growth, and thus the utilization rate of complex compounds were reduced^3,26^. In this study, the RDA analysis results showed that there was obvious difference in metabolism of soil microorganisms to exogenous carbon sources between without N fertilizer input, chemical fertilizer alone and organic manure treatments, indicating that organic manure treatments has greatly changed the soil environment, heterogenous organic manure and microorganisms were introduced, and then significantly changed the utilization characteristics of soil microorganisms to exogenous carbon sources.

In the present study, the soil microbial growth rate were increased, but the soil microbial CUE were decreased under application of organic manure condition. These results were also confirmed in the previous research by using MicroResp^TM^ method, the soil microbial metabolism ability to exogenous carbon (release to CO_2_) were significantly increased with organic manure practices^27^. In addition, the metabolic capacity of soil microorganisms to exogenous carbon sources in non-rhizosphere soils were higher than that of rhizosphere soils (Fig. 2), which was inconsistent with the results of soil basic respiration based on ^18^O-H_2_O method. The reason may be that MicroResp^TM^ method mainly monitors microbial decomposition of exogenous carbon, and it doesn’t differentiate between excitation effects, but ^18^O-H_2_O method mainly monitors microbial decomposition of soil carbon. On the other hand, rhizosphere soils were adaptability to exogenous carbon source input (root exudates) conditions. Meanwhile, the positive stimulation effect of decomposition of original organic carbon in non-rhizosphere soils were induced with input of exogenous carbon sources, and thus microbial mineralization of organic carbon were enhanced^26^.

## Conclusions

In this study, carbon source utilization characteristics in rice rhizosphere and non-rhizosphere soils under different short-term manure N input rate management conditions using a novel substrate-independent method were performed. The results indicated that characteristics of carbon source utilization in rhizosphere and non-rhizosphere soils were significantly affected by the short-term manure N input rate managements in a double-cropping rice paddy field. In summary, our results indicated that application of organic manure practices promotes soil microbial biomass carbon content and soil microbial growth rate in both rhizosphere and non-rhizosphere soils, whereas application of inorganic fertilizers promotes soil microbial carbon utilization efficiency. The results also showed that application of organic manure practices reduces carbon utilization efficiency in both rhizosphere and non-rhizosphere soils. The metabolic capacity of soil microorganisms to exogenous carbon sources in non-rhizosphere soils were higher than that of rhizosphere soils with different fertilizer treatments. And the utilization rate of carboxylic acids, amino acids and carbohydrates by soil microorganisms were increased with application of organic manure managements. There is an obvious difference in characteristic of carbon source metabolism between application of inorganic fertilizers and organic manure treatments. However, future studies were needed to investigate how changes of carbon source utilization soil microbial structure under different manure N input practices influence on ecological functions of rhizosphere microorganism.

## Materials and methods

### Sites and cropping system

The experiment was begun in 2017. It was located in Ningxiang County (28°07′N, 112°18′E) of Hunan Province, China. At beginning of this experiment, the surface soil characteristics (0–20 cm) were as follows: pH 6.80, soil organic carbon (SOC) 22.75 g kg^−1^, total N 2.24 g kg^−1^, total phosphorous (P) 0.66 g kg^−1^, total potassium (K) 14.45 g kg^−1^, available N 178.90 mg kg^−1^, available P 18.45 mg kg^−1^, and available K 69.50 mg kg^−1^. The climate condition (annual mean precipitation and evapotranspiration, monthly mean temperature) of this region, soil types and soil texture, and crop system were described by Tang *et al*.^13^.

### Experimental design

The experiment including five fertilizer treatments: (1) 100% N of chemical fertilizer (M0), (2) 30% N of organic manure and 70% N of chemical fertilizer (M30), (3) 50% N of organic manure and 50% N of chemical fertilizer (M50), (4) 100% N of organic manure (M100), and (5) without N fertilizer input as control (CK). There were three replications and each plot size was 88.0 m^2^ (10.0 m × 8.0 m). The M0, M30, M50 and M100 treatments received the same total amount of N, phosphorus pentoxide (P_2_O_5_), potassium oxide (K_2_O) (the total amount of N, P_2_O_5_, K_2_O were included chemical fertilizer and that from organic manure) during early rice and late rice growth period, respectively. During early rice and late rice growing season, the applied total amount of N were 135.0 and 165.0 kg ha^−1^ (60%, 30% and 10% at basal, tillering and full heading stages), 54.0 and 45.0 kg ha^−1^ of P_2_O_5_, 67.5 and 90.0 kg ha^−1^ of K_2_O, respectively. All the P_2_O_5_ and K_2_O fertilizers were applied at tillage and then transplanting rice seedling. During early rice growth period, the total quantity of organic manure added to paddy field for the M30, M50 and M100 treatments were 828.0, 1380.0 and 2760.0 kg ha^−1^, respectively. During late rice growth period, the total quantity of organic manure added to paddy field for the M30, M50 and M100 treatments were 1012.5, 1687.5 and 3375.0 kg ha^−1^, respectively. During early rice and late rice growing season, the N, P_2_O_5_ and K_2_O content of organic manure were 48.9 g kg^−1^, 17.3 g kg^−1^, and 15.1 g kg^−1^. Before transplanting rice seedling, organic manure were added to paddy field and incorporated into 0–20 cm soil layer with tillage.

### Soil sampling and sample preparation

Soil samples were collected at tillering stage of late rice in 2018. The rhizosphere and non-rhizosphere soils were collected and preprocessed according to the method described by Tang *et al*.^13^. Briefly, rhizosphere soil was operationally defined as soil adhering to the total roots after gentle shaking. The whole plant with their roots were extracted from soil and after shaking off the loosely adhering soil, the tightly adhering soil (i.e. rhizosphere soil) was carefully collected. In order to obtain the enough rhizosphere soil for multiplicating, twenty plants were randomly selected from each plot, and these rhizosphere soils were pooled to form one composite sample. Non-rhizosphere soil was defined as unvegetated soil adjacent to the rice plants. The unvegetated soil cores (5 cm diameter) adjacent to the rice plants (i.e. non-rhizosphere soil) were sampled at depth 0–20 cm. Correspondingly, one composite non-rhizosphere soil consisting of twenty cores was taken from each plot. Thus, three composite samples of rhizosphere and non-rhizosphere soils with each fertilizer treatment were collected at sampling time. The fresh samples were placed immediately on ice box and transported to the laboratory. Plant roots were removed by passing the sample through a 2-mm mesh sieve, and aliquots of the samples were then stored at room temperature until soil chemical properties analysis (for pH, C/N, NH_4_^+^-N, NO_3_^−^-N, SOC, and total nitrogen (TN)), at −20 °C until molecular analysis the types of exogenous carbon source (carboxylic acids, amino acid, carbohydrate, complex compounds). The samples were pre-incubated at 15 °C in aerated polyethylene bags for a total duration of six days before the beginning of the incubation, during which soil microbial carbon utilization efficiency (CUE) and microbial biomass turnover was determined. The pre-incubation was performed to allow the respiration to reach basal rate after sieving. Since the soils were at approximately 45% field capacity the soil water content was not manipulated.

## Soil laboratory analysis

### Soil chemical properties analysis

Soil chemical properties were measured according to the methods described by Bao^28^ and Wu *et al*.^29^. Briefly, soil pH was measured with a compound electrode (PE-10, Sartorious, Germany) using a soil to water rate of 1: 2.5. SOC and TN content were determined by an elemental analyzer (Carlo Erba 1110, CE Instruments) coupled to a Delta Plus isotope ratio mass spectrometer (Finnigan MAT) via a Conflo III (Thermo Fisher)^28^. Soil NH_4_^+^-N and NO_3_^−^-N concentrations in the extracts were determined by flow injection analysis^28^. Soil microbial biomass carbon (MBC) and microbial biomass nitrogen (MBN) contents were determined by chloroform fumigation-extraction method^29^.

### Determination of soil CUE

After the pre-incubation period, soil microbial CUE were determined based on incorporation of ^18^O from ^18^O-labeledwater into microbial genomic DNA following Spohn *et al*.^9^. Briefly, the respiration flux (C_Respiration_) were calculated based on the amount of carbon dioxide (CO_2_)-C produced during the incubation and the duration of the incubation period. The flux of C allocated to biomass production (C_Growth_) were calculated by dividing the amount of microbial biomass carbon produced during the incubation by the duration of the incubation. It should be noted that production of microbial biomass carbon (growth) does not necessarily imply a net change in the pool size (net growth)^9^.

Based on the steady-state assumption, the amount of C taken up by the soil microbial biomass (C_Uptake_) were calculated as

1 $${{\rm{C}}}_{{\rm{Uptake}}}={{\rm{C}}}_{{\rm{Growth}}}+{{\rm{C}}}_{{\rm{Respiration}}}$$

where C_Growth_ was the flux of C allocated to biomass production (growth), and C_Respiration_ was the flux of C allocated to the production of CO_2_ (respiration).

Soil microbial CUE were then calculated by the following equation^9^.

2 $${\rm{CUE}}=\frac{{{\rm{C}}}_{{\rm{Growth}}}}{{{\rm{C}}}_{{\rm{Growth}}}+{{\rm{C}}}_{{\rm{Respiration}}}}$$

### Characteristics of utilization of different types of exogenous carbon sources

The MicroResp^TM^ approach as described by Campbell *et al*.^27^ were used to measure exogenous carbon sources (carboxylic acids, amino acid, carbohydrate, and complex compounds), with the modifications described in detail in Lalor *et al*.^6^ and Banning *et al*.^7^. Briefly, the indicator dye with the gel detector plate consisted of 20 ppm cresol red dye, 240 mM potassium chloride and 4 mM sodium bicarbonate set into a 1% gel of noble agar (150 μL per well). Soil (300 μL total volume) was added to the 96-well microtiter deep well plates after 30 μL of each substrate had been dispensed (three replicate wells per substrate plus nine water controls per plate). This gave a final average water content of 60% of water holding capacity (WHC). Substrates were supplied at 30 mg ml^−1^ soil water or at 7.5 mg ml^−1^ soil water for those not readily water soluble. Substrate solutions were adjusted to pH 5.5–6.0. Incubations were run for 4 h at 25 °C. Absorbance of the detector plates was determined using a microplate reader at 590 nm (ASYS Expert 96, Biochrom, UK)^6,7,27^. Microbial carbon metabolism was investigated according to the method described by Campbell *et al*.^27^. Briefly, based on the calibration curve of CO_2_ production rate and light absorption value of specific wavelength by indicator, the exponential attenuation model was fitted to calculate the metabolic capacity of soil microorganisms to different carbon sources (CO_2_ production rate) within 4 hours.

CO_2_ production rate [μg (g·h)^−1^] were then calculated by the following equation:

3 $${{\rm{CO}}}_{2}\,{\rm{production}}\,{\rm{rate}}\,=\frac{{{\rm{CO}}}_{2}\times 10000\times L\times 12\times 273}{22.4\times (273+T)\times M\times t}$$

where *T* was the incubation temperature (25.0 °C), *L* was the volume of per hole deep orifice plate (945 μL), M was dry weight of soil (g), and *t* was incubation time (4 h).

### Statistical analysis

The statistical analyses of this manuscript were conducted by using SAS 9.3 software package^30^. To study the relationship between soil chemical properties and soil microbial carbon source utilization rate, we used a redundancy analysis. The redundancy analysis were performed using “vegan” packages in the R v3.20 statistical environment. The data of different treatments means in this manuscript were compared by using one-way analysis of variance (ANOVA) following standard procedures at the *p* < 0.05 probability level.
